# TGF-β as Predictive Marker and Pharmacological Target in Lung Cancer Approach

**DOI:** 10.3390/cancers15082295

**Published:** 2023-04-14

**Authors:** Valeria Ramundo, Maria Luisa Palazzo, Elisabetta Aldieri

**Affiliations:** Department of Oncology, University of Torino, 10126 Torino, Italy

**Keywords:** lung cancer, TGF-β, marker, epithelial to mesenchymal transition, tumor development, metastasis

## Abstract

**Simple Summary:**

Lung cancer (LC) represents the leading cause of cancer incidence and mortality worldwide. LC is a lung tumor associated with genetic mutations and environmental (tobacco smoking) or pathological conditions, with poor prognosis and a difficult pharmacological approach. TGF-β is a molecule that regulates different biological processes at a pulmonary level, and its alteration has been associated with LC development and metastasis. Despite advancements in knowledge of the molecular mechanisms involved in LC, this tumor is still characterized by an unfavorable prognosis and current therapeutic options are unsatisfactory. Some studies have demonstrated that TGF-β overexpression could be considered a potential predictive marker in LC prognosis, and TGF-β inhibition has been shown to prevent LC metastasis. Moreover, TGF-β inhibitors could be used in combination with chemo- and immunotherapy, thereby improving patient survival. Overall, targeting TGF-β may be a valid possibility to fight LC, and is a novel and effective strategy against this aggressive cancer.

**Abstract:**

Lung cancer (LC) represents the leading cause of cancer incidence and mortality worldwide. LC onset is strongly related to genetic mutations and environmental interactions, such as tobacco smoking, or pathological conditions, such as chronic inflammation. Despite advancement in knowledge of the molecular mechanisms involved in LC, this tumor is still characterized by an unfavorable prognosis, and the current therapeutic options are unsatisfactory. TGF-β is a cytokine that regulates different biological processes, particularly at the pulmonary level, and its alteration has been demonstrated to be associated with LC progression. Moreover, TGF-β is involved in promoting invasiveness and metastasis, via epithelial to mesenchymal transition (EMT) induction, where TGF-β is the major driver. Thus, a TGF-β-EMT signature may be considered a potential predictive marker in LC prognosis, and TGF-β-EMT inhibition has been demonstrated to prevent metastasis in various animal models. Concerning a LC therapeutic approach, some TGF-β and TGF-β-EMT inhibitors could be used in combination with chemo- and immunotherapy without major side effects, thereby improving cancer therapy. Overall, targeting TGF-β may be a valid possibility to fight LC, both in improving LC prognosis and cancer therapy, via a novel approach that could open up new effective strategies against this aggressive cancer.

## 1. Introduction

Tumors of the lung represent the leading cause of cancer incidence and mortality worldwide [1]. Lung cancer (LC), based on the type of the origin cell, is classified into small-cell lung (SCLC) or non-small-cell lung (NSCLC) cancer. The latter is further divided histologically into adenocarcinoma (50% of cases), squamous cell carcinoma and neuroendocrine cancer [1,2]. LC onset is strongly related to genetic mutations and environmental interactions, such as tobacco smoking, asbestos, radon, air pollution and arsenic, and also to pathological conditions, such as inflammation or chronic obstructive pulmonary disease (COPD) [1,3].

Despite advancement in knowledge of the molecular mechanisms involved in LC, this tumor is still characterized by an unfavorable prognosis, and the current therapeutic options for LC, including surgery, radiation therapy, chemotherapy and molecularly targeted therapy (e.g., epidermal growth factor receptor or anaplastic lymphoma kinase inhibitors and immunotherapy) [2,4] are unsatisfactory, resulting in a dismal 5-year survival rate and fatal outcomes of LC patients [5]. Therefore, improving overall survival for LC patients is the real challenge for research. Concerning pre-clinical research, it has been shown that transforming growth factor (TGF)-β plays a crucial role at the pulmonary level, both in physiological conditions, such as lung organogenesis, and in pathological ones, including tumor diseases such as LC [6,7]. In particular, this cytokine is the major driver of the epithelial to mesenchymal transition (EMT), a reversible physiological process involved in tumor onset, progression, and metastasis [8]. Growing evidence suggests the crucial importance of TGF-β and EMT in LC progression [4,9,10,11], and therefore, a greater understanding of the molecular mechanisms surrounding them could provide key predictive markers and therapeutic targets for LC.

In this review, we present an overview of recent studies regarding the role of TGF-β in driving LC development and/or metastasis via EMT events, in an attempt to provide a possible framework that can be used in the diagnostic/prognostic and therapeutic approaches against this aggressive cancer.

## 2. Lung Cancer: Molecular Mechanisms

In the lung, different risk factors may lead to the establishment of a favorable microenvironment for the tumor onset. Among these, are some non-modifiable and modifiable risk factors. The former risks include age, gender, race and family history; the latter one includes tobacco and cannabis smoking, asbestos, radon, air pollution, arsenic, infections (e.g., HIV or Tuberculosis) and COPD [1,12]. In particular, the modifiable risk factors can evoke a continuous loop between the injury of alveolar epithelial cells and persistent repairing and regenerative attempts, with consequent fibroblast activation, ECM (extracellular matrix) deposition and resulting structural alterations [13]. Overall, this results in a situation of chronic inflammation, characterized by endoplasmic reticulum stress and oxidative stress induction, mitochondrial dysfunction and apoptosis. In this inflammatory status, many modulators are released, such as interleukins (e.g., IL-1β, IL-13) or chemokines (e.g., chemokine ligand 2) [13]. This permanent inflammatory status can lead to pulmonary fibrosis and/or accumulating mutations, which could drive LC development [13]. In this generated tumor microenvironment, the tumor stroma includes different components, such as endothelial cells, ECM, infiltrating immune cells and cancer-associated fibroblasts (CAFs) [5]. During tumorigenesis, both infiltrating inflammatory and tumor cells release cytokines and growth factors that sustain tumorigenesis [14]; for example, it was observed that IL-6 and IL-8 are more strongly upregulated in squamous cell carcinoma than in adenocarcinoma of the lung, whereas TGF-β and IL-8 are relevant in pulmonary onset and metastasis [15]. Among these factors, TGF-β is particularly crucial; once produced and released by inflammatory cells (e.g., macrophages) or cancer cells in the tumor milieu [10,13,16], TGF-β acts on fibroblasts by transforming them into CAFs, which are important cells implicated in promoting tumor progression. It has been shown in resected squamous NSCLC tumor cells that MMP-2 CAFs expression is a negative prognostic sign [5] and, furthermore, high TGFβ levels in CAFs have been shown to be correlated to a negative regulation of growth and proliferation, and a better radiosensitivity and survival in SCLC [17]. In addition to TGF-β, reactive oxygen species (ROS), released by nicotinamide adenine dinucleotide phosphate 4 (NOX4) in LC cells, are also involved in LC tumorigenesis [10], thus contributing to the tumor microenvironment. Moreover, the oncogene Ras was usually demonstrated to be constitutively activated in LC through Ras or EGFR (EGF receptor) mutations, playing an important role in LC development, mainly via TGF-β [16,18].

In tumorigenesis, autocrine and paracrine secretion of the above-mentioned factors contribute to the promotion of tumor development and notably, among these, TGF-β is vital in supporting the creation of a malignant microenvironment and a consequent drug resistance [14], via a specific signaling pathway.

## 3. TGF-β Signaling Pathway in Cancer

The TGF-β superfamily, evolutionarily conserved [11], includes approximately 40 structurally and functionally related members, which are involved in different cellular events, including morphogenesis, embryonic development, inflammation and cancer [6], and exerts its functions through a set of receptors, such as the TGF-β type I (TGFβRI) and type II (TGFβRII) receptors [19]. TGF-β carries out its functions by stimulating apoptosis and survival, cell differentiation, EMT, regulating the expression of non-coding RNAs and the maintenance of stemness [18,20].

TGF-β is secreted and stored in the ECM as inactive large precursor polypeptides (latent TGF-β) composed of three segments: the N-terminal signal peptide, which is important in its secretion; a large precursor segment known as the latency-associated peptide (LAP); and the C-terminal TGF-β monomer peptide, which forms the mature dimeric TGF-β [20]. When the LAP is cleaved from the mature TGF-β peptide by a furin protein convertase, it is noncovalently linked to the mature TGF-β dimer and forms the small latent complex, which then binds to other proteins and forms the large latent complex, which, in turn, links to latent TGF-β binding proteins (LTBPs). The latent TGF-β complex is also activated proteolytically by ROS [18]. The active form of TGF-β binds its autophosphorylated transmembrane serine/threonine kinase receptor, TGFβRII, which in turn transphosphorylates TGFβRI by its kinase domain [20]. Interestingly, the expression of TGFβRI and TGFβRII proteins is implicated differently in various subtypes of LC; in particular, TGFβRII seems to be downregulated in poorly differentiated lung adenocarcinomas and squamous cell carcinomas [19]. Interestingly, TGFβRI overexpression in LC is associated with poor overall survival [7], and it could represent a crucial component of the complex by controlling downstream EMT signaling pathways, mainly via Smad and non-Smad pathways [20]. In the first pathway, TGFβRI interacts with and phosphorylates Smad2 and Smad3 [21]. Thereafter, they are released from the receptor complex and bind Smad4, forming the activated Smad complex, which migrates into the nucleus where some genetic transcription factors [3,20] involved in cell proliferation are regulated. Although the Smad pathway is the main pathway for canonical signaling, other downstream pathways exist and are known as non-Smad pathways. These pathways include: the stress-activated kinases p38 and JNK (Jun N-terminal Kinase), the mitogen activated protein kinase (MAPK), the extracellular-signal-regulated activated kinases 1 and 2 (ERK1 and ERK2), Rho GTPases, mTOR and the phosphoinositide 3-kinase (PI3 K)/Akt pathways. These non-Smad pathways can synergize with the Smad one in different processes, such as cell growth inhibition or, particularly, EMT [20].

Among the non-Smad pathways that are well known to be in conjunction with the Smad one, are the JNK and p38 MAPK signalling pathways; in particular, the obligatory requirements for TGF-β-induced JNK/p38 activation are the TGF-β-activated kinase 1 (TAK1), which activates and is activated by TGF-β signaling, and the ubiquitin ligase TNF receptor (TNFR)-associated factor 6 (TRAF6) [21]. The latter binds the TGF-βR complex, activating the RING finger E3 ligase and lysine-63 (K63)-linked polyubiquitination of TRAF6 itself, allowing the TAK1 binding and activation, which in turn acts on the JNK/p38 pathways [21].

Interestingly, recent studies highlight how the cleavage and nuclear accumulation of TβRI are selectively involved in TGF-β pathway activation in tumors, including prostate, breast and lung carcinomas, suggesting that there is another non-Smad mechanism that is activated by TGF-β cytokines. According to this mechanism, TGF-β, through the ubiquitin ligase TRAF6, leads to TβRI polyubiquitination and its cleavage by TNF-α converting enzymes (TACEs), in a PKCζ-dependent manner [22,23]. After TβRI cleavage, its intracellular domain (ICD) is released and binds p300 to promote the transcription of tumor-related genes, such as Snail [23]. In this regard, the TGF-β induced nuclear accumulation of TβRI ICD and increased invasiveness in the A549 cells was observed, in a TACE- and PKCζ-dependent manner [22]. Moreover, TRAF6 allows the association of Presenilin 1 (PS1), a γ-secretase, to the TβRI complex, its polyubiquitination, and, consequently, its cleavage and activation, a mechanism explored in in vitro models of LC [23]. Moreover, TGF-β is known to active the phosphoinositide 3-kinase (PI3K)-Akt signaling pathway, which is crucial in tumorigenesis, via the recruitment of both TβRII and TβRI [24].

Therefore, in tumorigenesis, TGF-β sustains, via Smad and/or non-Smad pathways, tumor progression, and regulates different downstream effectors in tumor cells, thus contributing to the composition of their microenvironment and constituting, in this way, a crucial point in driving carcinogenesis [14].

## 4. TGF-β Molecular Mechanism in Lung Cancer

**Figure 1:**
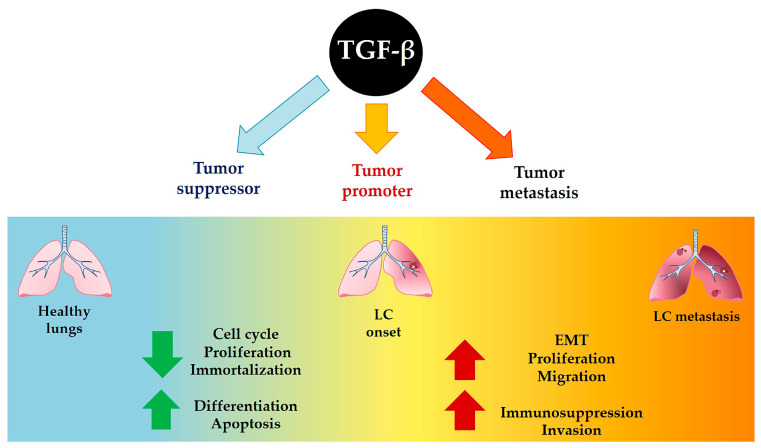
TGF-β functions in LC development and metastasis.

It is widely known that TGF-β plays a biphasic role in tumorigenesis [3,20]; in the early stages of tumor development, TGF-β acts as a tumor suppressor, inhibiting cell cycle progression and proliferation, preventing cellular immortalization and promoting cellular differentiation or apoptosis. However, a basic molecular mechanism of this switch between TGF-β as a tumor suppressor and TGF-β as a tumor promoter has yet to be clarified [25]. In the later stages of tumorigenesis, TGF-β acts as a tumor stimulator, thereby promoting cellular changes associated with migration, invasion and metastasis, immunosuppression, angiogenesis, myofibroblast generation, interaction between cancer cells, extracellular matrix and EMT [26] (Figure 1).

In addition, the increase of TGF-β in many tumors regulates the epithelial plasticity of stromal cells, contributing to cancer progression [18]. Thus, TGF-β is strongly related to tumorigenesis and the tumor microenvironment onset in mesenchymal stem cells and cancer-associated fibroblasts, and it is a cytokine expressed by both stromal and tumor cells [5,14]; it has been demonstrated its overexpression induces the progression in advanced solid tumors, thus representing an indicator of a poor prognosis [18]. Moreover, TGF-β overexpression induces drug resistance by the regulation of the immune system, resistance to antiangiogenic therapies by promoting tumor vascularization, and resistance to immune checkpoint blockades [14].

As described above, TGF-β is a cytokine implicated in different molecular processes and, among these, it is included in a particularly important event called epithelial to mesenchymal transition (EMT), of which TGF-β is considered the main driver. As demonstrated, both TGF-β and EMT are deeply involved in LC onset and progression at the molecular level.

### TGF-β and EMT in Lung Cancer

The EMT is a physiological, dynamic, and reversible process involved in differentiation, development and wound healing [16,27], during which epithelial cells lose junctions, polarity and the expression of epithelial markers (such as E-cadherin and β-catenin), and acquire a fibroblastic phenotype, migratory and invasive capacities and the expression of mesenchymal markers (such as N-cadherin and vimentin) [8,28]. Although EMT is a physiological event, it also contributes to a few pathological conditions including fibrosis, cancer and metastasis [9]. The EMT process is regulated by EMT transcription factors (EMT-TFs), including Snail1, Snail2, twist-related proteins (Twist) and zinc finger E-box-binding homeobox 1 and 2 (Zeb 1/2), which modulate the expression of epithelial and mesenchymal markers, thus promoting EMT [8,28].

TGF-β is the major driver of EMT and promotes tumor onset, invasion and metastasis through EMT induction, and, consequently, tumor malignancy [29], via Smad or non-Smad pathways [3]. In Smad pathways, TGF-β induces downstream expression of EMT-TFs, whereas in non-Smad pathways, TGF-β controls EMT via MAPK/ERK1/2, NF-κB/Snail, JAK/STAT3 and PI3K/AKT pathways [3]. TGF-β, via both these pathways, stimulates EMT in different cancers, thereby promoting tumor invasion and metastasis [30]; one of the mechanisms mediated by TGF-β promotes the generation of CAFs from epithelial cells in tumor stroma through EMT [18].

The regulation of TGF-β signalling in lung cancer occurs also at the post-translational level. One of the EMT-TFs, Snail, is strongly regulated at the post-translational level. In particular, Snail degradation is controlled by phosphorylation by GSK-3β kinase action [31], polyubiquitination and, thereafter, proteasome degradation [32]. Another regulating post-translational mechanism is the acetylation mediated by CREB-binding protein (CBP), that acts on Snail, thereby activating itself [32]. Moreover, in LC, the p300, a histone acetyltransferase, knockdown negatively affects Snail transcription, representing a correlation between Snail and p300 [33]. Among the EMT-TFs activated downstream by TGF-β signalling pathways, Snail is a crucial factor involved in cancer invasiveness and stemness, and is affected by various post-translational mechanisms. Both polyubiquitination and sumoylation control Snail activation; the former drives EMT via TRAF6 [22,32], while the latter one drives EMT via the conjugation of SUMO proteins at the Snail K234 residue. Both modifications regulate post-translational EMT-TF Snail, thus controlling EMT events and other pro-tumorigenic cellular processes [32].

TGF-β also affects EMT via microRNAs (miRNAs) and long non-coding RNAs (lncRNAs) regulation [3]. These small non-coding RNAs can affect different cellular processes [3], including TGF-β-induced EMT, which can be regulated by some lncRNAs and miRNAs. TGF-β-induced EMT-associated miRNAs are upregulated in LC, including miRNA-93, -128-3p, -9, -134, -487b, -330-3p, -1246, -9-5p, -181b-5p and -23a; the long non-coding RNAs are downregulated, including, miRNA-132, -203, -145, -205, -124, -422a, -196b, -940, -22, -200 family, -149, and -497 [3]. In addition, LINC00858 is a lncRNA that regulates some tumor-related miRNAs, via MAPK and TGF-β signaling pathways, and its abnormal expression is associated with prognosis, clinical stage and metastasis [34]. Therefore, EMT is associated with the modulation of miRNAs expression; in particular, miRNA-330-3p and miRNA-205 were found upregulated and downregulated, in NSCLC cell lines and tissues, respectively, and miRNA-330-3p inhibitor or miRNA-205 mimics have been demonstrated to control TGF-β-induced EMT in NSCLC cells [35]. Furthermore, miR-330-3p promoted cell invasion and metastasis in NSCLC, may by induce EMT and miR-205, which could restrain NSCLC by suppressing EMT [35]. Moreover, miRNA-16 is involved in pulmonary tumorigenesis, particularly in lung adenocarcinoma, where transcription factor AP-2α (TFAP2A) has been shown to be overexpressed and to induce EMT via miR-NA-16 family/TFAP2A/PSG9/TGF-β [36].

In addition to regulation by non-coding RNAs, TGF-β can exert its influence on EMT by other effectors; G-protein regulator signaling 6, a tumor suppressor protein that negatively regulates TGF-β-induced EMT in NSCLC, and its low expression was associated with poor survival, which suggests that it is a possible LC prognostic marker [37]. In addition to QKI-5, an important protein involved in the RNA signal transduction, it has been shown that this protein decreased in metastatic lung adenocarcinoma, and its overexpression inhibits TGF-β-induced EMT-mediated invasion and metastasis via TGFβRI [38]. Furthermore, LIM and SH3 domain protein 1 (LASP1), considered as new biomarkers of metastasis, interact on the TGF-β pathway via regulating phospho-Smad2/3 and Snail1 localization, thereby modulating the expression of EMT markers (e.g., vimentin, N- and E-cadherin) [39].

Intriguingly, the TRAF6-TAK1-JNK/p38 pathway also plays a very important role in TGF-β-induced EMT; indeed, both p38 inhibition and TRAF6 knockdown can prevent EMT-related changes, thus playing a crucial role in TGF-β-induced EMT via the TRAF6-TAK1-p38 pathway [21]. Moreover, it has been observed that TRAF6-mediated polyubiquitination of the TβRI intracellular domain can directly regulate both EMT target genes (Vimentin, Twist1 and N-cadherin) and cyclin D1 expression levels in prostate cancer cells, thus strongly suggesting its implications in TGF-β-induced EMT [40].

Interestingly, an integrated analysis of the immune landscape in NSCLC through EMT scores demonstrated that EMT was associated with a significantly lower infiltration of CD4 T-cells in lung adenocarcinoma, CD4/CD8 T-cells in squamous cell carcinoma and increased immunosuppressive cytokines, including TGF-β [41]. Moreover, proteomic analysis of alveolar type II (ATII) epithelial cells treated with 4-OHT or TGF-β showed that RAS and TGF-β activation induces a total and partial EMT, respectively [16]. Kim et al. [29] reported that in human normal lung epithelial (BEAS-2B) and human lung cancer (A549) cell lines, TGF-β can promote EMT and cancer stemness acquisition, activating Slug and CD87 by their promoter demethylation [29]. Recently, it was also observed that in LC, EMT induction could be an important outcome of nickel exposure and has been associated with LC development [9].

In a dose-dependent manner, TGF-β or, alternatively, ginsenosides Rk1 and Rg5 treatment in NSCLC A549 cells, regulate EMT by suppressing the Smad and NF-kB/ERK pathways (non-Smad pathway) [42]. In A549 cells, ginsenoside CK prevents TGF-β-induced EMT and metastasis [43]; furthermore, in TGF-β-treated A549 cells, a novel compound called *ent*-caprolactin C inhibited the phosphorylation of Smad2/3 and suppressed the EMT cell marker proteins, so that TGF-β-induced EMT was inhibited, constituting a potential antimetastatic agent [44]. In addition, TGF-β induced the reprogramming of the amino acid metabolism, necessary to EMT in NSCLC, which depends on prolyl 4-hydroxylase α3 (P4HA3), an enzyme implicated in cancer metabolism; if downregulated, this promotes TGF-β-dependent changes in amino acids inhibition and EMT, and consequently tumor metastasis occurred [45]. Finally, in NSCLC cell lines (A549 and SPC-A1), TGF-β-activated the SMAD3/4 complex by positively regulating N-cadherin [46]. Moreover, SH2B3 was decreased while TGF-β was elevated in LC, promoting cancer cell anoikic resistance, invasion and EMT, via JAK2/STAT3 and SHP2/Grb2/PI3K/AKT signaling pathways [47]. Another interesting protein is the formin-like 1 (FMNL1) protein, which is overexpressed in NSCLC and was detected in bone metastasis; in FMNL1-knockdown A549 and PC9 cells and in mice, the metastatic, migratory and invasive properties of cells are reduced via TGF-β/SMAD-mediated EMT inhibition [48]. Moreover, the high mobility group protein A2 (HMGA2) plays an interesting role, as HMGA2 competes with TGF-β type III receptors for the let-7 miRNA family, thus driving EMT in inducing cancer metastasis at the pulmonary level [49]. Interestingly, it has been demonstrated that EMT inhibition can also improve the sensitivity to EGF receptor-targeted therapy in LC patients [50].

Considering the pulmonary district more widely, Turini et al. [28] showed, that human mesothelial cells incubated with TGF-β or asbestos fibers, a risk factor of LC and malignant pleural mesothelioma development, underwent EMT via the Smad-dependent pathway and its downstream effectors, highlighting the action of TGF-β as a mediator in inducing EMT [28]. A recent report demonstrated that the fibroblast growth factor 2 (FGF2) synergically enhanced TGFβ-induced EMT in human lung epithelium cell line BEAS-2B, maintaining TGFβ-induced morphologic changes, and increased the migration of TGFβ-treated cells [51]. Instead, concerning the non-Smad pathway, it was observed that NF-κB regulates the ROS-mediated EMT process by activating Snail transcription factor in A549 cells [10]. Thus, the inhibition of NF-κB, prevented the increase of NOX4 expression and TGF-β induced ROS and EMT in A549 cells, confirming the TGF-β-mediated EMT process via NF-κB/NOX4/ROS signaling pathway [10].

Taken as a whole, this growing evidence suggests that TGF-β-mediated EMT is a well-characterized process involved in LC and implicates different components that could contribute to LC development and metastasis.

## 5. TGF-β in Lung Cancer Development and Metastasis

### 5.1. TGF-β in Lung Cancer Development

TGF-β regulates a variety of biological processes, and its presence is physiological and necessary for lung morphogenesis and homeostasis; its alteration is strongly associated with pulmonary fibrosis and, in particular, LC progression [13]. LC cells secrete TGF-β, but the malignant behavior results in a loss of its tumor suppressor effects, such as reduced expression and inactivation of TGF-β or its receptors, with the consequent loss of the TGF-β inhibitory effect on proliferation. In LC, TGF-β overexpression is associated with better prognosis in 5-year patient survival [27]. An important body of evidence exists and supports the crucial implications of TGF-β in LC; indeed, the TGF-β-induced target gene expression and the consequently driven EMT in lung adenocarcinoma cells, promote the constitutive activation of the Ras oncogene through its mutations or EGFR (EGF receptor), thus synergizing with the EMT induced by TGF-β [18]. The importance of the role of EGFR in this mechanism is also supported because TGF-β-mediated IL-6/JAK/STAT3 signaling promotes erlotinib resistance in EGFR mutant NSCLC models [14]. In addition, in NSCLC, when EGFR is altered, phosphorylated STAT3 is overexpressed, so the JAK/STAT3 signaling axis triggers survival, proliferation and resistance to the EGFR inhibitors [14]. Furthermore, TGF-β-induced EMT in LC is boosted by the action of the TNF-α or IL-1β of the tumor microenvironment [18].

EMT is an event strongly related to a variety of pathogenic conditions, such as pulmonary fibrosis associated with the change in ECM [32], also called Type-II EMT [52]. This cellular process occurs as a response to injury, but the precise mechanism is still to be clarified [53]. However, emerging studies seem to investigate its role in different disorders; for instance, the TGF-β1-induced EMT could provide the relevant quantity of fibroblasts in idiopathic pulmonary fibrosis (IPF), a chronic lung disease, characterized by morphologically abnormal alveolar epithelial cells (AEC), procoagulant factors and fibrogenic cytokines [53]. This progressive condition could lead to LC onset, in particular when associated with angiogenesis (Type-III EMT) [52]. Interestingly, the activation of EGFR–RAS–ERK axis in human alveolar epithelial type II (ATII) cells induces ZEB1-mediated EMT, which is involved in lung fibrosis via paracrine signalling, including the expression of tissue plasminogen activator (tPA), to underlying fibroblasts [33]. In addition, intact lung epithelial layers suppressed fibroblast proliferation and matrix deposition, whereas AEC exposed to TGF-β change their morphology in a fibroblast-like phenotype, as the consequence of EMT induction [53].

TGF-β exerts various functions and its genetic sequence variations are associated with modification of TGF-β production and/or activity, which also affects individual susceptibility to LC; it was observed that people with at least one −509T allele have a decreased risk of lung adenocarcinoma and SCLC, and, at the same time, the 869T > C polymorphism and the combined TC + CC genotype were associated with a decreased risk of SCLC compared with the TT genotype [26]. In LC development, different TGF-β-related proteins can contribute to sustain, potentiate or inhibit molecular pathways in which TGF-β is involved. TGF-β-induced factor homeobox 2 (TGIF2) phosphorylation triggered the EGFR–RAS–ERK signaling pathway to enhance the stemness of lung adenocarcinoma cells, thus promoting its progression; by silencing TGIF2, there is a decrease of cancer stem cell-like properties in NSCLC cells [54]. Moreover, REGγ supplies the TGFβ-Smad signaling pathway with the ubiquitin-ATP-independent degradation of Smad7 (inhibitor of the TGFβ pathway) [55]. In addition, the overexpression of AlkB homolog 5 (ALKBH5), an RNA N6-methyladenosine (m6A) demethylase, suppresses TGF-β-induced EMT and invasiveness of NSCLC cells, reducing the mRNA stability of TGFβR2 and SMAD3, but increasing the mRNA stability of SMAD6 [56]. Moreover, NKX2-1 is an important lung epithelium-specific regulator of EMT; in A549 cells, this protein is upregulated and inhibits TGF-β induced EMT by interrupting the Smad3/4 complex formation in the nucleus [18]. However, in SCLC, the TGF-β signaling pathway is mainly inactivated; in fact, mutations in genes involved in this pathway are uncommon [18], so the tumorigenic role of TGF-β is much more crucial and relevant in NSCLC than SCLC.

**Figure 2:**
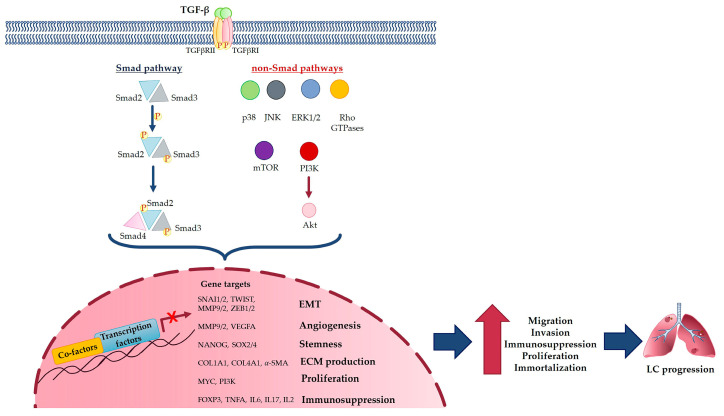
TGF-β signaling pathway in LC development and metastasis.

Take as a whole, the TGF-β signaling pathway is widely involved in LC development, as shown in Figure 2.

### 5.2. TGF-β in Lung Cancer Metastasis

TGF-β is involved in the spread of tumors in the body, playing a critical role in promoting metastasis [25], and the inhibition of TGF-β has been demonstrated to prevent metastasis in various animal models [18]. Khan et al. [46] observed that when NSCLC cells, pretreated with TGF-β to induce EMT, were injected into mice models, these cells tended to spread more than non-treated cells [57]. Moreover, the occurrence of the persistent infectious status is related to tumorigenesis in LC; notably relevant is the correlation between human immunodeficiency virus (HIV) infection and LC risk and progression [1]. It was highlighted that HIV infection, in NSCLC patients, is associated with a poorer prognosis, and the loss of Tat-interacting protein 30 (TIP30) is related to metastasis via the promotion of TGF-β-induced EMT, invasion and stemness; the loss of TIP30 seemed to promote Snail nuclear translocation [58]. Interestingly, in 3D collagen-based matrices systems, high concentrations of collagen and TGF-β may drive the formation of NSCLC cell spheroids and promote invasiveness, demonstrating the crucial role of the microenvironment composition in supporting metastasis [59]; however, the presence of TGF-β seems to be an independent risk factor for the occurrence of metastasis in NSCLC [15]. In addition to the direct role of TGF-β, the Hedgehog signaling pathway is also linked indirectly to the TGF-β effect and correlated to a poor prognosis and aggressiveness in NSCLC, via Oxy210, an oxysterol-based dual inhibitor of both pathways. Oxy210 suppresses EMT, proliferation and invasion, thus enhancing the toxicity of carboplatin in NSCLC A549 cells [60]. Moreover, TGF-β regulates clonogenicity in lung adenocarcinoma cell line, protects against stress-induced apoptosis and increases adhesion, spreading, lung retention and metastatic outgrowth [7]. Additionally, the TGF-β pathway specifier LRG1 (leucine-rich alpha-2-glycoprotein 1) has been demonstrated to be overexpressed in endothelial cells via STAT3, and seems to be involved in lung metastasis [61].

Regarding proteins that can affect the TGF-β signaling pathway, Du et al. [54] showed the possible correlation between high levels of TGIF2 expression and lymph node metastasis in patients with lung adenocarcinoma. Additionally, results showed that mice bearing TGIF2-silenced xenografts develop smaller tumors and fewer lung metastases [54]. Moreover, it has been shown, by transplanting cell tumors into nude mice, that the lysine-specific histone demethylase 1 (LSD1) demethylates the SEPT6 promoter, thus positively regulating Septin 6 (SEPT6), which, through the TGF-β/Smad pathway, accelerates metastasis in NSCLC [62]. In addition, it was investigated in in vivo experiments that the overexpression of cellular prion proteins drives the invasion and metastasis of the LC, an effect linked to TGF-β and PD-L1, which is important for the role of regulatory T cells in LC and immunotherapeutic approach [63]. Furthermore, TGF-β is the major driver of pleural dissemination in metastatic LC; in particular, pleural mesothelial cells that co-cultured with A549 cells expressed high levels of TGF-β and underwent apoptosis and senescence [64]. Interestingly, molecules inhibiting the metastatic effect mediated by TGF-β were discovered; among these crizotinib, a tyrosine kinase inhibitor, blocks TGF-β signaling by abrogating Smad pathway in an ALK/MET/RON/ROS1-independent manner in NSCLC cells [65], thereby inhibiting migration, invasion and metastasis. However, bergamottin, a furanocoumarin, has also been shown in LC cells to inhibit TGF-β-induced EMT, invasiveness and multiple oncogenic cascades, such as PI3K/Akt/mTOR, thus showing its strong antimetastatic potentiality [66].

As described above, the TGF-β signaling pathway is widely involved in LC metastasis, as shown in Figure 2.

## 6. Targeting TGF-β in Lung Cancer Approach

### 6.1. TGF-β as Predictive Marker in Lung Cancer

**Figure 3:**
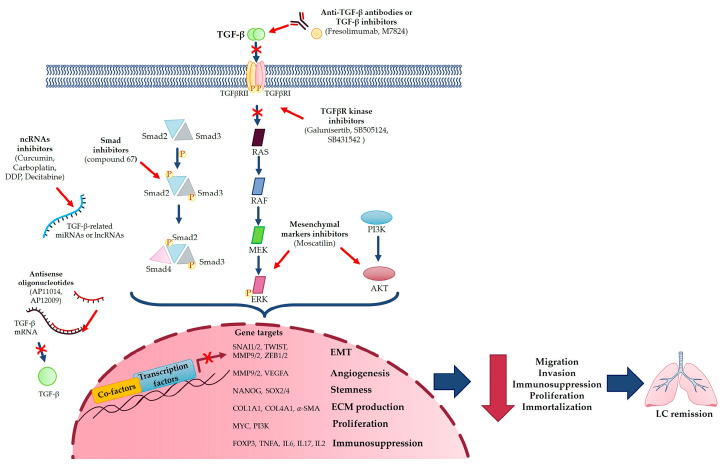
Targeting TGF-β signaling pathway in LC.

Despite the current approaches for LC treatment, the identification of novel predictive markers is needed to obtain an early diagnosis and provide patients with a better prognosis. For this reason, TGF-β would lend itself well, as demonstrated by Diego de Miguel-Perez et al. [67], which found that high levels of TGF-β in extracellular vesicles were associated with nonresponse to immune-checkpoint inhibitors, shorter progression-free survival and overall survival, resulting in higher accuracy than the current tissue PD-L1 marker [67]. Li et al. [68] demonstrated that significant levels of TGF-β expression in patients were associated with poor survival; thus, TGF-β expression can predict a significantly worse prognosis for patients with LC [68]. Moreover, the TGF-β-inducible protein (TGFBI), an extracellular matrix component, and the methylation of its promoter, is relatable to NSCLC malignancy, thus constituting a possible predictive marker for NSCLC prognosis [69]. Interestingly, it seems that TGF-β gene overexpression may contribute to an inherited predisposition to LC; the TGF-β −509C > T and 869T > C polymorphisms, and their haplotypes, were significantly associated with a risk of LC [26]. Moreover, miRNAs and lncRNAs can also be used as biomarkers to predict outcomes for LC patients [3]. Furthermore, Gordian et al. [70] showed that the TGF-β-EMT signature may be considered as a potential predictive biomarker, as they successfully discriminated lung cancer cell lines undergoing TGF-β-induced EMT and predicted metastasis-free survival in lung adenocarcinomas [70]. Taken together, this evidence shows the significant possibility of using TGF-β as a predictive marker in improving LC diagnosis and prognosis (Figure 3).

### 6.2. TGF-β as Pharmacological Target in Lung Cancer

As far as the therapeutic approach to LC is concerned, it should be kept in mind that, currently, LC is a thoracic neoplasm that is difficult to cure and that develops resistance to therapy. The establishment of drug resistance is a particular problem for the efficacy of immune, targeted and cytotoxic therapies [14,71]. As previously reported, TGF-β signaling is aberrant in different tumors, such as LC, helping tumor progression, metastasis and the development of resistance to therapies [14]. Therefore, targeting different levels of the TGF-β pathway could provide novel attractive therapeutic options to treat the disease more satisfactorily. In this context, a useful therapy could be based on the following:

- TGF-β inhibitors, such as Fresolimumab, a TGF-β-specific human monoclonal antibody [14];
- Blocking TGF-β production via antisense oligonucleotides to directly target TGF-β, for example TGF-β1 antisense oligonucleotide (AP 11014) [20,25] and TGF-β2 suppressor (AP 12009) [20,27];
- TGF-β-induced EMT inhibitor (compound 67) that specifically inhibits migration and invasion, blocking Smad2 phosphorylation or some EMT markers, such as MMP-2 and MMP-9 [30];
- Targeting Smad6, which could reduce tumor growth via a protein which promotes LC survival [27];
- Using a bifunctional TGF-β ligand trap such as M7824, a fusion protein that simultaneously targets both PD-L1 and TGF-β [72];
- TGF-β receptor kinase inhibitor, such as Galunisertib, a molecule kinase inhibitor [25], or the small molecule ATP competitive compounds SB-505124 and SB431542, which inhibit the enzymatic activity of TGF-β RI type receptor, thereby preventing TGF-β-induced EMT [27];
- Targeting miRNAs and lncRNAs involved in TGF-β-mediated LC progression with different compounds, such as curcumin, carboplatin, DDP or Decitabine [3], and, moreover, exploiting the inhibitory effects of miRNA-454-3p against TGFB2 and miR-454-3p on NSCLC, which reversed TGF-β overexpression, thus providing a promising strategy for treating NSCLC [73];
- Blocking TGF-β-induced EMT with different molecules, such as Bufalin or CX-4945, direct inhibitors of TGF-β-induced EMT, or Moscatilin, an inhibitor of mesenchymal markers, via suppressing ERK and Akt signaling pathways [2].

Taken as a whole, targeting TGF-β could be a good possibility at the therapeutic level, particularly because, at the time of the diagnosis, tumors already overcome the growth-inhibiting effects of TGF-β (Figure 3).

A wide body of evidence has shown the existence in nature of different products capable of lung cancer chemoprevention [74,75,76,77,78,79]. It is well known that some of these natural derivates exhibit an antagonizing effect against TGF-β activities in lung cancer, as is the case of piperine, an amide derived from the Piper spices, which inhibits the TGF-β1 induced EMT, ERK 1/2 and SMAD 2 phosphorylation in A549 cells [74]. In addition to piperine, the *Duchesnea indica* treatment of A549 cells co-incubated with TGF-β1 and decreased TGF-β1 induced-vimentin expression level, thereby counteracting TGF-β1 induced EMT [75]. Likewise, kaempferol, a natural dietary flavonoid, exerts a negative effect on TGF-β1-induced EMT and cell migration in A549 cells by inhibiting the Akt1-mediated phosphorylation of Smad3 [76]. Interestingly, tannic acid, a natural dietary polyphenolic compound, added to TGF-β1 in pre-incubated A549 and BEAS-2B cells, has been shown to inhibit TGF-β1-induced EMT, cell proliferation, Smad proteins phosphorylation and Akt, ERK1/2, JNK1/2 and TGF-β receptors through a direct interaction between TGF-β1 and tannic acid [77]. Moreover, TGF-β1-induced EMT development in A549 cells can also be inhibited by resveratrol, a natural polyphenolic compound that enhances E-cadherin expression and decreases fibronectin, vimentin, Snail1 and Slug one, thus preventing TGF-β1-induced cell adhesion, migration and invasion [78]. Another natural compound is a lignan called arctigenin that has been demonstrated to inhibit TGF-β-induced migration, Smad2/3, ERK phosphorylation and Snail expression in human NSCLC cells [79]. Taken together, these studies propose new natural TGF-β-blocking products and encourage the discovery of new ones to counteract LC progression.

Recently, CRISPR/Cas9 technology has been used to explore the possible role of different proteins in TGF-β-induced EMT events in LC [80,81]. For instance, the depletion of Ras Association Domain Family 10 (RASSF10) in TGF-β-induced EMT in different cell lines (e.g., small, non-small and squamous cell LC) induces cell growth and invasiveness after TGF-β treatment [80]. Similarly, CRISPR/Cas9 technology has been used to study the role of GABARAPL1, an autophagy-related gene, during TGF-β/TNF-α-triggered EMT in LC and kidney adenocarcinoma cell lines [81]; in LC cells, KO GABARAPL1 occurred during the induction of EMT via a defective GABARAPL1-mediated autophagic degradation of the SMAD proteins, proposing the possible role of GABARAPL1in a negative EMT-regulatory loop [81]. This evidence suggests that the loss of RASSF10 or GABARAPL1 could contribute to lung tumor progression by supporting TGF-β-induced EMT [80,81].

Although targeting the TGF-β pathway could be helpful, especially in combinatorial therapy, hostile effects could not be verified since TGF-β executes a huge number of functions, particularly in relation to the immune system [14]. Fortunately, no alterations have been documented, as it is a situation with abnormally high levels of TGF-β and these types of therapies seem provide a good compromise between maximum therapeutic effect and minimum side effects [14]. Indeed, the inhibition of TGF-β signaling in vivo has been shown to block tumorigenesis [25] and TGF-β receptor inhibitors or specific TGFβ inhibitors have been shown not to elicit major side effects.

### 6.3. Targeting TGF-β in Lung Cancer Approach

The aim of this review was to focus on the importance of TGF-β as a potential diagnostic, prognostic and therapeutic LC approach. TGF-β was identified as a possible LC marker of interest in the preventive field and as a potential pharmacological target, thus paving the way for future clinical trials targeting TGF-βs with specific inhibitors in LC patients. Thus, the TGF-β signature, particularly at the late stage of LC, may be considered a predictive marker for improving LC prognosis, and TGF-β inhibition has been demonstrated to reduce tumor growth and prevent LC metastasis.

LC is a widespread tumor, and its high and rising incidence, together with the lack of effective therapies, underlines the urgent need for novel biomarkers to guide LC diagnostic workup and better identify LC patients prior to personalized treatment, thereby improving LC prognosis and therapeutic efficacy. Indeed, improving the overall survival of LC patients is the real challenge; targeting TGF-β, particularly in combination with chemo- and immunotherapy, will thus represent a starting point for future improvements to LC management, in the attempt to better overcome this difficult-to-treat tumor.

## 7. Conclusions

Overall, TGF-β inhibition may be a valid possibility to fight LC. TGF-β can be a good marker for LC, particularly in NSCLC, which can so be used to try to improve LC prognosis. It can also be used in LC pharmacological therapy, particularly in combination with chemo- and immunotherapy, a novel approach that could open up new effective strategies against this aggressive cancer.

## Data Availability

The data can be shared up on request.
